# Development of the Pharm‐SAVES educational module for gatekeeper suicide prevention training for community pharmacy staff

**DOI:** 10.1111/hex.13741

**Published:** 2023-02-28

**Authors:** Amanda N. Stover, Jill E. Lavigne, Alexis Shook, Catherine MacAllister, Wendi F. Cross, Delesha M. Carpenter

**Affiliations:** ^1^ Division of Pharmaceutical Outcomes and Policy University of North Carolina Chapel Hill North Carolina USA; ^2^ Department of Veterans Affairs Center of Excellence for Suicide Prevention Canandaigua New York USA; ^3^ Department of Pharmacy Practice, Wegmans School of Pharmacy St John Fisher College Rochester New York USA; ^4^ Department of Psychiatry University of Rochester Medical Center New York Rochester USA

**Keywords:** community pharmacy, gatekeeper training, suicide prevention

## Abstract

**Introduction:**

Pharmacists are one of the most accessible health professionals in the United States, who, with training, may serve as gatekeepers who recognize suicide warning signs and refer at‐risk individuals to care. Our objective was to codesign a 30‐min online gatekeeper training module (Pharm‐SAVES) specifically for community pharmacy staff.

**Methods:**

Over a period of 8 months, a nine‐member pharmacy staff stakeholder panel and the Finger Lakes (New York) Veterans Research Engagement Review Board each worked with the study team to codesign Pharm‐SAVES. Formative data from previous interviews with community pharmacists were presented to the panels and guided website development.

**Results:**

Four key topics were identified for brief skills‐based modules that could be delivered asynchronously online. To help pharmacy staff understand their opportunities as gatekeepers in suicide prevention, statistics and statements from the Joint Commission and pharmacy professional organizations were highlighted in Module 1 (‘Why Me?’). Module 2 (‘What can I do?’) presents the five gatekeeping steps (SAVES): (1) Recognize suicide warning **S** igns, (2) **A** sk if someone is considering suicide, (3) **V** alidate feelings, (4) **E** xpedite referral, and (5) **S** et a reminder to follow‐up. Module 3 (‘How does it work?’) provides three video scenarios modeling SAVES steps and two interactive video cases for participant practice. Module 3 demonstrates use of the 24/7 National Suicide Prevention Lifeline, including the DOD/VA Crisis Line. Module 4 (Resources) includes links to national resources and a searchable zip code‐based provider directory. Pharm‐SAVES was codesigned with pharmacy and veteran stakeholders to deliver brief, skills‐focused, video‐based interactive training that is feasible to implement in busy community pharmacy settings.

**Conclusion:**

Pharm‐SAVES is a brief, online suicide prevention gatekeeper training program codesigned by researchers, community pharmacy and veteran stakeholders. By actively engaging stakeholders at each stage of the design process, we were able to create training content that was not only realistic but more relevant to the needs of pharmacy staff. Currently, Pharm‐SAVES is being evaluated in a pilot randomized controlled trial for changes in pharmacy staff suicide prevention communication behaviors.

**Patient or Public Contribution:**

Stakeholder engagement was purposefully structured to engage pharmacy staff and pharmacy consumers, with multiple opportunities for study contribution. Likewise, the involvement of patient/public contribution was paramount in study design and overall development of our study team.

## 1 INTRODUCTION

Suicide is a persistent public health problem in the United States^1^ and globally.^2^ As many suicides can be prevented, multiple strategies focused on suicide prevention have been implemented over the past several decades. One mechanism for prevention is gatekeeper training programs, which teach community members to identify suicide warning signs and intervene with at‐risk individuals when signs are recognized.^3^ Training programs for gatekeepers may vary in structure and format, but the overall goal is to enhance participant knowledge, attitudes, and skills to identify at‐risk individuals and refer them to services.^4^, ^5^ Previous studies report that gatekeeper training as a preventative intervention for suicide can increase participant knowledge of suicide prevention, build prevention skills, and increase gatekeeper confidence to discuss suicidal behavior.^4^, ^6^, ^7^

Pharmacists are one of the most accessible health professionals in the United States^8^ and, with training, can serve as gatekeepers who recognize suicide warning signs and refer individuals for assistance.^9^, ^10^ However, community pharmacists have noted significant barriers to suicide prevention gatekeeping, including discomfort communicating about suicide, lack of time and privacy, and limited referral options for patients.^9^ Concise training to address these barriers has yet to be developed even though formative studies have documented community pharmacists' strong interest in participating in suicide prevention training.^9^, ^11^

Therefore, our objective was to work with stakeholders to codesign a 30‐min online training module specifically for community pharmacy staff. A codesign approach to research involving stakeholders has the potential to enrich the development, implementation, and evaluation of health interventions.^12^ Stakeholder engagement in the research process allows for more readily usable and seamless uptake of health care innovations.^13^ Codesign strategies are particularly well‐suited for interventions implemented in complex work systems such as community pharmacies where the intervention may be employed across a diverse group of recipients.^13^ Additionally, codesign strategies have been successfully implemented in suicide prevention campaigns. One such example is the codesign of a suicide prevention social media campaign in partnership with young people.^14^ This study demonstrated the feasibility to safely engage young people in codesign of a suicide prevention intervention. Feedback from young people engaged with researchers ultimately produced recommendations for youth suicide prevention campaigns and encouraged safe communication about suicide.^14^

In this paper, we describe the process for working with stakeholders to prioritize training content and interactive elements. Interventions may provide better outcomes and be better received when a stakeholder codesign approach is used to develop them.^15^

## 2 METHODS

A codesign approach was used to develop the suicide prevention training module. Vechakul and colleagues define codesign as a cooperative process bringing people and design professionals together to find new and innovative solutions for different problems of everyday life, this can include the innovation of technological products and/or management of complex social issues.^16^ Using this definition, the current project aimed to work with community pharmacy stakeholders, a group of veterans, suicide prevention experts, an educational design specialist, and team of website designers to address suicide prevention in community pharmacy settings. To further guide our approach, we adapted key concepts outlined by Goodman and Thompson for stakeholder engagement research^17^ and methods outlined by Donetto and colleagues.^18^ This project drew heavily from engaged participation methods where stakeholders held shared decision‐making authority.^17^ Additionally, meeting structure and approach was like the six stages of experience‐based codesign process outlined by Donetto et al. in 2015.^18^ Although Donetto and colleague's model was not followed identically, set‐up, stakeholder engagement, and codesign meetings followed a similar structure outlined by their process.

Two stakeholder panels were involved in reviewing material developed for Pharm‐SAVES: a community pharmacy stakeholder panel including nine community pharmacy staff (i.e., pharmacists, pharmacy technicians, and a student pharmacist) and the Finger Lakes VA Veterans Research Engagement Board (VREB). The VREB consisted of 10 veterans from all branches, service periods, races, and genders, including officers and enlisted members. In addition to soliciting input from the pharmacy stakeholders and the VREB panel, a website development, educational design, two suicide prevention, and a patient‐provider communication expert, who attended the pharmacy panel meetings, provided input throughout the course of module development.

Before meeting with all stakeholders, the research team, comprised of two primary investigators, a postdoctoral research assistant (postdoc) with previous experience in suicide prevention, and two study coordinators, met to discuss results from a previous formative study on pharmacy staff preferences for suicide prevention content. Data from the formative study showed that pharmacy staff preferred training that included: local suicide prevention referral resources, training that takes under 30‐min to complete, and incorporates three to four realistic role play scenarios, including a telephone interaction.^9^ The formative data also included previous interviews with community pharmacists addressing preferences for communication, content delivery, length of module, and types of video cases desired.^9^, ^11^, ^19^ This information was summarized and orally presented over Zoom teleconferencing to the pharmacy stakeholder panel. After receiving input from the pharmacy stakeholders on overall direction for the module, the study team met weekly to draft initial content. The module was programmed by the University of North Carolina (UNC) Eshelman School of Pharmacy MEDIA team, comprised of an education specialist, a videographer, a graphic designer, and a computer programmer.

Over the course of 8 months, 9 pharmacy stakeholder panel meetings were held in the late afternoon/evenings to accommodate busy pharmacy staff schedules (Table 1). For participants who could not attend a specific meeting, meeting minutes were distributed afterwards, and feedback on module content was obtained via email or phone. Additionally, topic materials and a meeting agenda were sent out the week before each meeting. The pharmacy stakeholders selected a recurring day and time that they were available to attend a monthly 1‐h meeting. The meeting occurred via Zoom, that had the option to call in for individuals who did not have reliable Internet access. During all meetings the meeting facilitator took notes and recorded meeting minutes in real‐time in a Word document. In addition to meetings with the pharmacy stakeholder panel, two meetings were held with the VREB panel, which reviews research to ensure applicability and usefulness to veterans. Meetings were facilitated by staff from the Center of Excellence for Suicide Prevention and the study postdoc through video teleconference. During both meetings, the VREB provided oral feedback on how to improve the written content, which is the text that would appear on the website, while the meeting facilitator took notes. Before the final stakeholder panel meeting, the website was reviewed by two suicide prevention experts with expertise in gatekeeper training. An outline of the meeting schedule and meeting content can be seen in Table 1.

**Table 1:** Stakeholder engagement meetings and content outline.

Meeting type	Number of attendees	Topics covered
Pharmacy Stakeholder Meeting 1	9	Reviewed the formative study outlining gatekeeper training needs of community pharmacy staff Watched existing videos modeling pharmacist‐ patient interactions of suicidal behavior Elicited specific feedback to determine if the interaction was realistic, an appropriate length, and needed to be adapted for pharmacy setting. Introduced VA SAVE, the suicide prevention gatekeeper training program developed by the VA Center of Excellence for Suicide Prevention (S.A.V.E. (psycharmor.org)).^11^ Finalized regular meeting time
Pharmacy Stakeholder Meeting 2	9	Reviewed scripts for the three proposed pharmacy video scenarios: (1) in‐person, (2) via telephone, and (3) drive‐through.^a^
VREB Panel Meeting 1	13	Reviewed proposed website content Reviewed scripts for the three proposed video scenarios, which take place: (1) in‐person, (2) via telephone and (3) at the pharmacy drive‐through.^b^
Pharmacy Stakeholder Meeting 3	11	Reviewed revised in‐person video script Presented written content for the training website Focused on structure of the website Website was divided into four key module topics: (1) Why me?, (2) What can I do? (Pharm‐SAVES), (3) How does it work? and (4) Referral resources.^c^
Pharmacy Stakeholder Meeting 4	10	Facilitated by the UNC MEDIA team Evaluated website layout and design o Three different website mock‐ups with varying layouts and interactive features.
VREB Panel Meeting 2	13	VREB reviewed the final drafts of video scripts and website mock‐up
Pharmacy Stakeholder Meeting 5	8	MEDIA team presented revised website with training content Solicited real‐time feedback on website modules for additional feedback Drafted interactive cases
Pharmacy Stakeholder Meeting 6	7	Presented the final version of the full training website Reviewed the Introduction page and Module 1 ‘Why me?’ content Feedback on Interactive Cases solicited, discussed the scripts and flow of content
Pharmacy Stakeholder Meeting 7	6	Reviewed:
		Updated Introduction page Module 2: ‘What can I do?’ (Pharm‐SAVES) Module 3: ‘How does it work?’ Module 4: Resources Summary page
Pharmacy Stakeholder Meeting 8	7	Completed review of:
		Module 2: ‘What can I do?’ (Pharm‐SAVES) Module 3: ‘How does it work?’ Module 4: Resources Summary page Added section with credit for contributors and checked credentials
Meeting with Suicide Prevention Experts	5	Study post‐doc presented Pharm‐SAVE website Solicited feedback on website content and structure Advised to make ‘Set a reminder to follow‐up’ a separate step
Pharmacy Stakeholder Meeting 9	7	Reviewed content proposed by suicide prevention expert Updated acronym to ‘Pharm‐SAVES’ based on feedback from suicide prevention experts Reviewed resource referral tool

^a^ The pharmacy stakeholder group was provided with the video scripts before the meetings to provide written feedback and scripts were then reviewed over Zoom in real‐time as a group.

^b^ The review of scripts was done via Zoom. The study post‐doc attended the meeting and took notes in real‐time which were typed and reviewed with the study team for integration into the revised scripts.

^c^ The content was outlined by the postdoc and reviewed by the study investigators. A written outline of content was provided to the stakeholders for review prior to the meeting.

Meeting minutes were recorded in a Word document in real‐time directly on the meeting agenda by either the study post‐doc or one of the study coordinators. Meeting minutes were reviewed weekly by the study team, where suggestions for website improvements, usability, module language, and content were extracted from meeting minutes. Any suggestions or edits related to website content (either written or video) were extracted and organized in a separate Word document. This Word document outlining website changes was then emailed to the UNC MEDIA team for incorporation into the course website.

Due to the sensitive nature of the topic material, stakeholders and project staff were provided with mental health resources. The study post‐doc was also trained in mental health and suicide prevention protocols and was available for debriefing or to provide appropriate referral and local crisis resources.

## 3 RESULTS

Twelve meetings occurred over the course of 8 months, during which there were nine meetings with an average of nine pharmacy stakeholders, two separate meetings with a veteran stakeholder (VREB) panel, and one separate meeting with two suicide prevention experts. Each meeting lasted roughly 1 h and was facilitated by the study post‐doctoral research assistant over Zoom. Using the written notes from the panel meetings, changes to website content were adopted iteratively and reviewed by the stakeholders over the course of the 8‐month meeting period.

The overall structure of the website was divided into four main sections (or modules): (1) ‘Why me?’, (2) ‘What can I do?’ (Pharm‐SAVES), (3) ‘How does it work?’ and (4) Resources. Stakeholders wanted content that modeled realistic pharmacy interactions to increase the pharmacy staff member's confidence to engage in suicide prevention gatekeeping behaviors. Stakeholders wanted to know how they could integrate SAVES into their pharmacy workflow with minimal disruption to prescription filling, counseling, and other pharmacy services. The four modules were decided upon after the pharmacy stakeholder panel was provided with a summary of the formative data,^9^ where they decided which barriers that were identified should be prioritized for the module. Module 2, ‘What can I do?’ was modeled after the VA SAVE training, which teaches the trainee to recognize the warning signs (S) of suicide, ask (A) the person directly if they are considering suicide, validate (V) feelings to encourage open communication, and Expedite (E) referral to treatment. Feedback on the steps of SAVE and previous video versions of SAVE training were obtained during the formative studies.^9^, ^11^, ^19^, ^20^, ^21^ During the pharmacy stakeholder panel meetings, panel members confirmed that they liked the steps of SAVE and recommended minimal changes because the steps were already highly applicable in the pharmacy environment.

**Figure 1:**
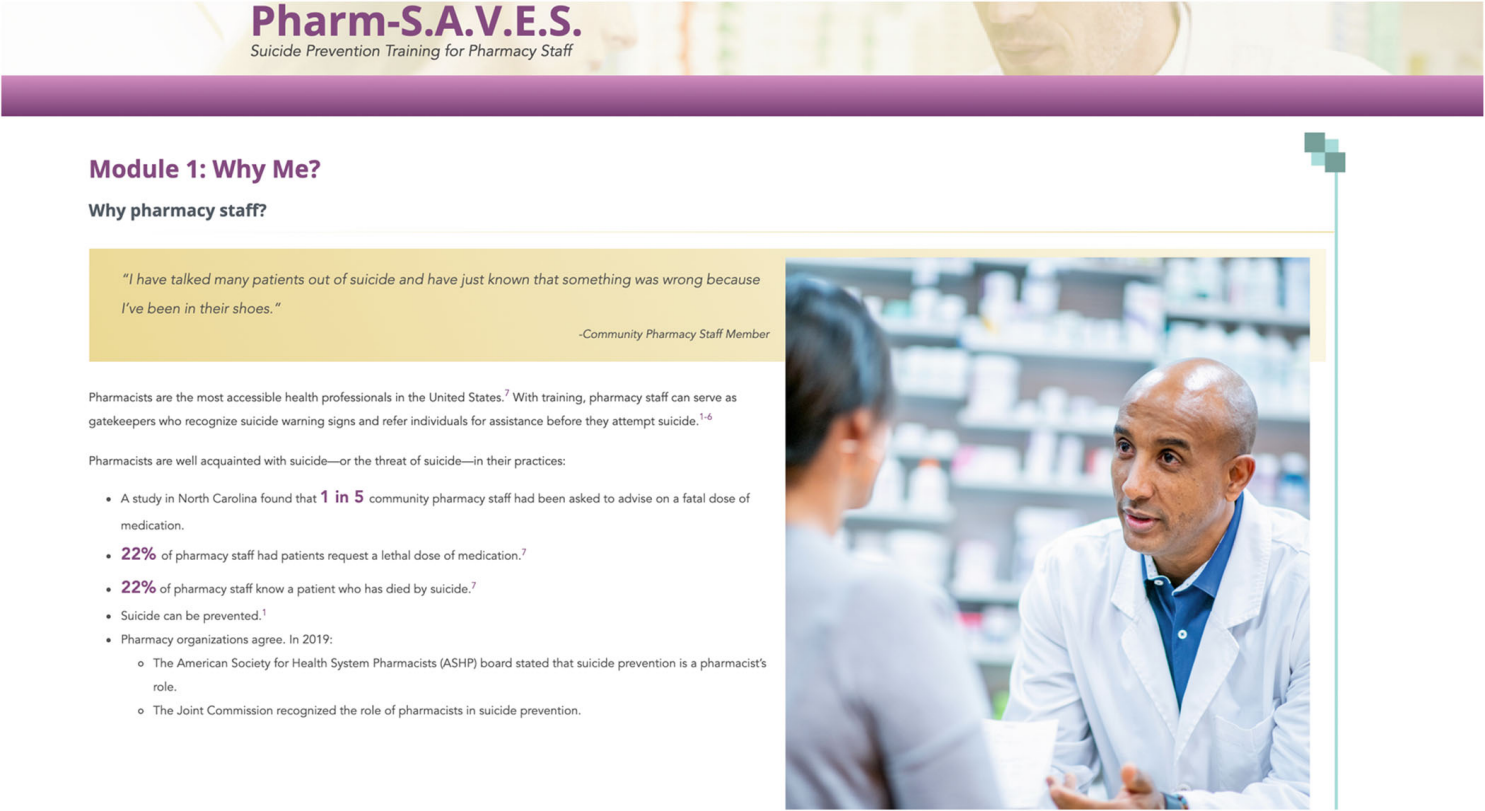
Pharm‐SAVES Module 1: Why Me?

One issue consistently raised by the pharmacy stakeholders was the importance of developing content that maximizes pharmacy staff ‘buy‐in’ and ‘relatability’. Therefore, the module starts with a section on ‘why me?’. Pharmacy stakeholders thought this section would help pharmacy staff (i.e., pharmacists and pharmacy technicians) understand the critical role they have in suicide prevention (Figure 1). They also wanted statistics on the likelihood that pharmacy staff may encounter a patient at risk of suicide and information about medications and suicide, which provides an overview of medications labeled for suicide risk and percentage of suicides carried out by poisoning. Stakeholders suggested including a section on medication and suicide because this directly pertains to their work as pharmacy staff. Lastly, there was a strong desire from pharmacy stakeholders to include a section about suicide statistics in the United States and suicide rates by gender and ethnicity because they wanted to know how suicide impacts people across genders, races, and ethnicities.

**Figure 2:**
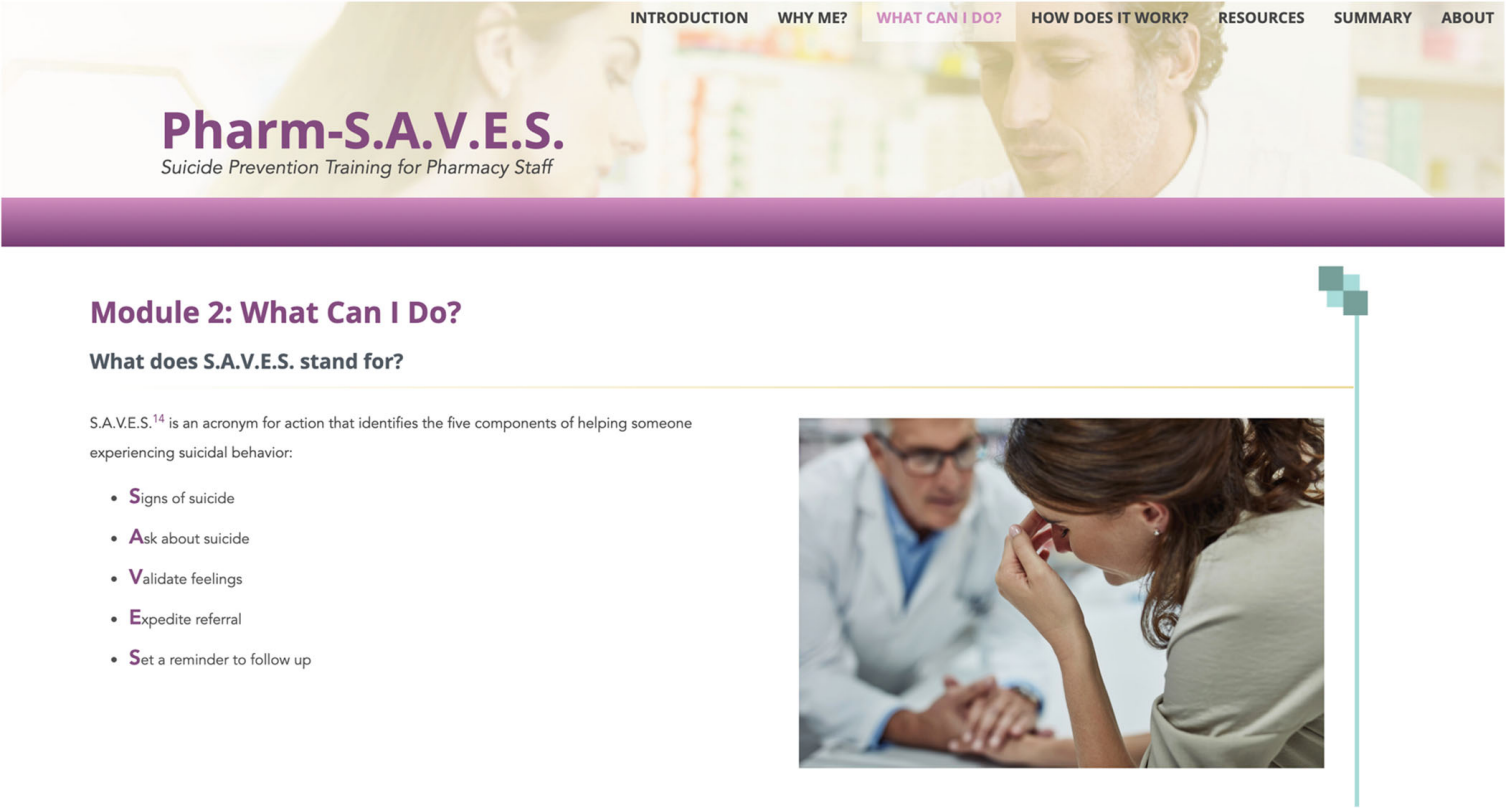
Pharm‐SAVES Module 2: What Can I Do?

Module 2 presents the acronym ‘SAVES’, which stands for: (1) Recognize the warning **S** igns, (2) **A** sk if someone is considering suicide, (3) **V** alidate feelings to encourage open communication, (4) **E** xpedite referral to resources, and (5) **S** et a reminder to follow‐up. Based on stakeholder panel feedback, a tab for each letter of the acronym was constructed to review each step (Figure 2). Pharmacy stakeholders liked how the acronym modeled simple and concise steps for action that could easily be integrated into a busy pharmacy workflow. Each tab includes quotes from practicing pharmacy staff to help participants connect with the information and provide a real‐world example of how to implement SAVES. The final ‘S’ was added after consulting with suicide prevention experts (Table 1) and is described later.

Under the first ‘S’ tab (“**S** igns of suicide”), the module reviews verbal and behavioral warning signs and signs of an immediate emergency to help pharmacy staff recognize suicide warning signs so they can then ask about suicide. Pharmacy stakeholders emphasized that the signs should be ones that could be recognized in the pharmacy setting and wanted example quotes of pharmacy staff who had observed these signs in practice. This was done to help facilitate recognition of warning signs since detailed assessments are not within the normal scope of pharmacy practice. Thus, this section also states that some patients may not show any signs of suicide and that the behaviors listed are meant to serve as a guide for identifying potentially at‐risk individuals. The ‘A’ tab (‘**A** sk about suicide’) encourages staff members to ask patients if they are thinking about suicide when they recognize suicide warning signs. Pharmacy staff emphasized how this was a very uncomfortable question and how video examples need to put content from this tab into action, specifically capturing the awkwardness of asking. In the ‘V’ (‘**V** alidate feelings’) tab, the module reviews how to accept and support disclosure of the patient's feelings and support help‐seeking. Then, the ‘E’ tab (‘**E** xpedite referral)’ emphasizes use of the National Suicide Prevention Lifeline (NSPL). Pharmacists cannot leave the pharmacy while on duty, so a lot of time was spent figuring out a feasible referral strategy for the pharmacy setting. The pharmacy stakeholders collectively agreed that referrals to the NSPL were feasible and could be integrated into community pharmacy workflow.

The ‘E’ tab reinforces two key points: (1) Patients with warning signs of suicide should not be left alone, and (2) In addition to local resources, the NSPL is open 24/7, 365 days and is free and confidential. Finally, the second ‘S’, or ‘**S** et a reminder to follow‐up’ tab, encourages pharmacy staff to set a reminder to follow‐up with the patient the day after the encounter. This step was added after meeting with two suicide prevention experts. These experts emphasized the importance of follow‐up contact, as it reinforces social support and validation of the at‐risk patient's feelings. Previous studies have equally shown that suicide prevention interventions that include a follow‐up component can reduce suicidal behavior^22^, ^23^ and increase treatment engagement.^22^ Therefore, it was suggested that follow‐up should be a separate step and not combined as part of the referral step.

Video examples ground the ‘how does it work?’ section, modeling drive‐through, telephone, and in‐person cases. Each scenario models a ~3‐min initial interaction and a 30‐s follow‐up (set a reminder to follow‐up) interaction. The drive‐through interaction was deemed particularly important to model given the increased use of the drive‐through during the COVID‐19 pandemic.^24^ The pharmacy stakeholder panel wanted these interactions to model a medication overdose (telephone scenario) and a situation where signs of suicide were not obvious (in‐person scenario). The scenarios also show a patient whose warning signs include problems affording essential medication (drive‐through scenario; Table 2). Additionally, VREB panel feedback was of particular importance during the creation of these scenarios to be relatable to veteran populations. During the videos, each step of the SAVES process includes text at the bottom right corner so that individuals know when the pharmacy staff are implementing each step. The suggestion to add the steps to the video was made by the educational designer and enthusiastically agreed upon by all stakeholders.

**Table 2:** Summary of video scenarios.

Scenario	Description
Drive‐Through	Presentation (Darnell): First‐generation college student. He has student health insurance and works multiple jobs to pay his own tuition. Darnell pulls up to the pharmacy drive‐thru window, clearly in a rush. He already appears to be on edge and somewhat agitated. He approaches the window cash in hand, eager to end his interaction as quickly as possible.
	Warning signs: Darnell Finds out that his insurance has changed and that he can no longer afford his inhaler. The pharmacist notices that he has become agitated and asks Darnell if he is ok. Darnell states that he is having a rough time, his bills are piling up, and that sometimes it would be easier if he just stopped trying.
In‐Person	Presentation (John): Reflects demographics of highest suicide death rate cohort of (Veterans): White, Male, Middle‐aged, Gulf War veteran. relatively clean shaven, above average education. Somewhat overweight, hypertension, and some musculoskeletal pain. Has insomnia, which is associated with suicide risk. Mild mannered/even courteous.
	Warning signs: John approaches the pharmacy counter to pick up his prescription. The technician asks John if he has any questions for the pharmacist. When asked, John replies with “Hey, yeah, is this enough that I won't have to wake up in the morning?”
Telephone	Presentation (Joanne): Joanne is a 45–55‐year‐old white female, recently divorced; living alone. She presents with a flat affect. Joanne calls the pharmacy asking about a recent prescription for a new sleep medication, zolpidem (Ambien) solemn and has little inflection in her voice.
	Warning signs: Joanne calls the pharmacy to ask the pharmacist a question about sleeping pills. At first, she is hesitant and asks the pharmacist what would happen if she overslept or if she wakes up in the middle of the night. However, eventually Joanne asks if she takes too many could she fall asleep and not wake up. This prompts the pharmacist to ask Joanne if that is something she is considering, and Joanne admits that this is something she is thinking about.

The pharmacy stakeholder panel felt it was important to keep users engaged and give opportunities to practice SAVES, which is why we developed the two interactive cases. The interactive cases allowed participants to practice SAVES in a controlled environment that provided feedback on their responses. One case depicts a white, middle‐aged male patient ‘Mr. Jones’ who is having difficulty sleeping. This demographic has a relatively high risk of suicide and is representative of the veteran population. A second case depicts an upbeat, female patient ‘Ms. Smith’ who asks about how much it would take to overdose on her medication. In each case, the trainee watches the video and types of responses to several questions, which are interspersed throughout the video. Example questions include: ‘Does Mr. Jones show any signs of suicide?’ or ‘How confident are you that you can ask Ms. Smith about suicide?’. Questions are a combination of free text response and multiple choice, where the participant is provided feedback after their response is submitted.

**Figure 3:**
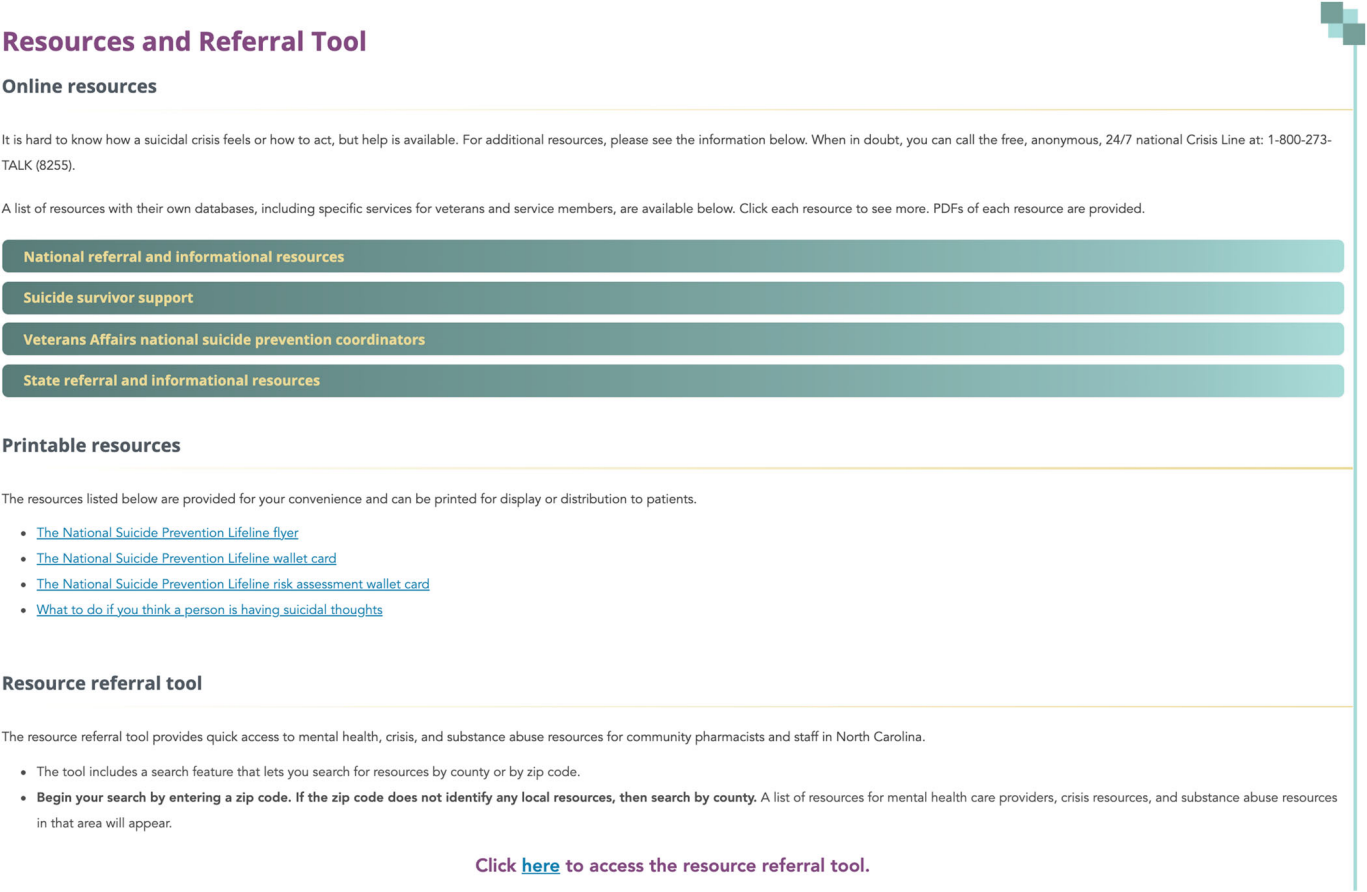
Pharm‐SAVES Resources and Referral Tool.

In addition to the NSPL, the pharmacy stakeholder panel also wanted a resource referral tool that provided a list of local behavioral health, substance use, and other resources. Using online searchable databases, including the Substance Abuse and Mental Health Services Administration (SAMHSA) treatment locator and the Veterans Administration (VA) treatment locator, a list of mental health and substance use treatment facilities, providers, and other resources were compiled by zip code and county. Resources were classified based on services provided, current location, and contact information. This tool provides a consolidated list of local resources that can be sorted by county or zip code to provide pharmacy staff with suicide prevention or crisis resources (Figure 3). Each resource included was checked for accuracy by the study post‐doc and a member of the pharmacy stakeholder panel. This included confirming working web‐links and cross‐checking phone numbers and address information.

## 4 DISCUSSION

Codesign approaches that directly involve stakeholders, have previously been shown to enhance the development and implementation of health interventions to improve patient outcomes across healthcare settings.^12^, ^25^ Through an iterative engagement process, our interdisciplinary study teams took a codesign approach to create suicide prevention training for community pharmacy staff. By working with stakeholders, both community pharmacists and the VREB panel, we were able to garner real‐world examples to model both the video content and interactive scenarios. Overall, the research team found that by actively engaging stakeholders at each stage of the design process, we were able to create training content that was not only realistic but more relatable to the needs of pharmacy staff. Furthermore, by reflecting on the codesign process, it is possible to gain a better understanding of its strengths and limitations.

### 4.1 Strengths and limitations

There were several strengths in taking a codesign approach for the development of suicide prevention training for pharmacy staff. One such strength was that a codesign approach helped provide a framework in which feedback from one session guided and informed future meetings. Although a general meeting schedule and broad topic outline were created at the beginning of the project, allowing for adaptation of topic material based on participant feedback was useful in module development. Information gathered in each session, could be evaluated by the study team, and helped inform the development timeline, module content, and set benchmarks in the development process to reach the overall end‐goal. This is a similar approach taken by other codesign driven pharmacy interventions^13^ and was equally helpful in our design process.

Since meetings were conducted virtually, it allowed more stakeholders to attend despite their busy work schedules. Likewise, the pharmacy stakeholders were from different areas of NC and were able to participate, which allowed for a dynamic view of pharmacy experiences from across the state of NC. Furthermore, virtual meetings allowed for the inclusion of the VREB panel organized out of the VA in New York, which allowed us to gather veterans' perspectives. Additionally, Zoom teleconferencing afforded the study team an opportunity to interact with suicide prevention experts nationally who provided critical input about module content without the need for costly travel or delays due to COVID‐19. All these factors helped result in a more comprehensive product that included input from pharmacy stakeholders who represent the target user, considerations from the VREB panel who represent potential recipients of the intervention as pharmacy consumers, and expert guidance that improved content accuracy and best practices.

Another key to success was having a consistently scheduled meeting time planned one month in advance. Pharmacy staff are quite busy, so the advanced scheduling promoted good attendance. At the first stakeholder meeting, a monthly meeting time was decided on. And the study postdoc sent a reminder 1 week before each meeting. Additionally, all stakeholders were emailed a meeting agenda in advance, and meetings were kept to 1 h. Having an agenda and a time limit helped keep the study team, and stakeholders focused and increased productivity.

Despite the overall success of our approach, this process was not without challenges. One challenge was soliciting feedback on written documents via email. In general, stakeholders were more responsive during in‐person meetings. Overall, the amount of written feedback received on documents was low. Even when given multiple days or weeks to review documents, very few stakeholders provided written feedback to emailed documents. This usually resulted in review of documents during the virtual meetings. Given low engagement with emailed feedback, an alternative approach that may have been more successful would be to provide mock‐ups of these documents during virtual meetings. Although this may have increased the number of total meetings, it may have ultimately been more efficient.

Another challenge we faced was receiving timely feedback. In addition to stakeholders, we also sought feedback from suicide research experts. Due to the time it takes to build a website and develop content, we did not seek outside expert input until near completion of the development process. This led to several iterations of edits that delayed website completion. In the future, including external suicide prevention education experts earlier and more frequently in the process would be beneficial.

To further evaluate the impact of the codesign process and effectiveness of the suicide prevention training, the online module described here (Pharm‐SAVES) is currently being evaluated in a randomized controlled trial. The purpose of randomized control trial is to examine whether it improves the suicide prevention knowledge, gatekeeper self‐efficacy, and communication behaviors of pharmacy staff. Additionally, information from the results of this trial will be used to improve the training module and expand access to additional community pharmacy settings to continue to build a useable, up‐to‐date training resource.

## 5 CONCLUSIONS

Pharm‐SAVES is a brief, online suicide prevention gatekeeper training program for community pharmacy staff who were integral to its development. The module incorporates written, video, and interactive content to maximize the participant's learning experience. By engaging with stakeholders to compose this content, pharmacy staff may be more likely to identify with module content and find the information more relevant and useful to their daily workflow. Successful codesign of the module was facilitated using zoom meetings and integration of stakeholder feedback on module content. Future codesign projects aimed at suicide prevention in community pharmacy settings would benefit from examining the needs of pharmacy consumers both with and without lived experience in mental health and suicide.

## AUTHOR CONTRIBUTIONS

**Amanda N. Stover**: Writing—original draft, review, and editing; conceptualization; project administration; resources. **Jill E. Lavigne**: Writing, review and editing; conceptualization; supervision; investigation; funding acquisition. **Alexis Shook**: Writing, review and editing; conceptualization; resources. **Catherine MacAllister**: Writing, review and editing; conceptualization. **Wendi F. Cross**: Writing, review and editing; conceptualization. **Delesha M. Carpenter**: Writing, review and editing; conceptualization; supervision; investigation; funding acquisition.

## CONFLICT OF INTEREST STATEMENT

The authors declare no conflict of interest.

## Data Availability

Data are available on request from the authors.
